# Supplementary material for: The Severity of Chronic Cough Diary (SCCD): development and content validation of a novel patient-reported outcome instrument for evaluating the symptom experience of chronic cough
Source: J Patient Rep Outcomes. 2023 Jul 10;7:65. doi: 10.1186/s41687-023-00605-8 (PMC10333155; doi:10.1186/s41687-023-00605-8)
Supplement: Supplementary file 1 — Supplementary Material 1 [file 41687_2023_605_MOESM1_ESM.docx]

**Supplementary Information**

**Supplementary methods**

***Stages 1–3 of Severity of Chronic Cough Diary development***

In Stage 1, a targeted literature review of the EMBASE database and USA and European Union clinical trials registers focused on peer-reviewed publications and instruments related to cough severity and frequency. The aim of the literature review was to understand the patient perspective of refractory chronic cough (RCC) to identify the psychometric properties of existing patient-reported outcomes (PROs) and their availability for potential use in RCC clinical studies.

In Stage 2, interviews were conducted with three clinical experts who treat patients with RCC in the UK, Ireland, and the USA, and one regulatory consultant with experience of developing PRO strategies for the evaluation of RCC and interaction with the US Food and Drug Administration on the topic. The aim of these interviews was to identify symptoms and impacts most relevant from the clinician and regulatory perspective.

At Stage 3, items in the first version of the Severity of Chronic Cough Diary (SCCD; Version 0.1) were generated in line with principles outlined by the International Society for Pharmacoeconomics and Outcomes Research PRO Good Practice Task Force [1, 2]. Draft item wording and conceptual coverage were informed by a review of published qualitative research and existing PRO instruments used to evaluate the severity and impact of cough. The items of the SCCD Version 0.1 were grouped to form a preliminary conceptual framework (Additional File 1: Supplemental Fig. S1).

***Full inclusion criteria for the Stage 4 qualitative research study***

1. Adults ≥18 years of age.
2. Participant self-reported diagnosis/history of chronic cough (lasting for at least 8 weeks) that has persisted for at least 1 year as refractory (unresponsive to treatment options) or idiopathic (unexplained)^a^.
3. Resident of the USA, the UK, or Germany^b^.
4. Able to understand, read, and speak English or German^b^ sufficiently to complete all assessments.
5. Willing and able to participate in an in-person or telephone interview which will be audio recorded and transcribed.
6. Willing and able to provide written informed consent.

^a^Physician confirmation of RCC diagnosis was not an eligibility requirement; however, on a voluntary basis, a clinician-reported clinical form collected medical information from US participants only. Questions included clinician report of RCC diagnosis, duration of RCC, etiology, and treatment history.

^b^Only US and UK interviews were conducted to ensure representation from the USA and Europe; Germany was included as an additional option in the protocol, in the event of difficulty recruiting patients from the UK; however, there were no interviews conducted in Germany.

***Full exclusion criteria for the Stage 4 qualitative research study***

1. Ongoing or past participation in any interventional clinical study within the past 4 weeks.
2. Smoking history within the past year.
3. Respiratory tract infection within the past 8 weeks.
4. Participant reports having significant concurrent airway diseases, including pneumonia, cancer, and chronic bronchitis with productive cough, chronic obstructive pulmonary disease, tuberculosis, or other active and/or chronic pulmonary infection.
5. Participant report of cough associated with heart problems; congestive heart failure; or angiotensin-converting enzyme inhibitors.
6. Participant has any other clinically relevant medical condition, including but not limited to severe comorbid condition, severe mental illness, visual impairment, substance abuse, or cognitive impairment which, in the opinion of the investigator, would interfere with participating in an interview and/or completing the study procedures.

**Supplementary results**

***Stages 1–3: Preliminary SCCD conceptual framework and instrument development***

In Stage 1, the targeted literature review identified 32 PRO instruments used to assess patient experiences of RCC. Of those, 15 were cough-specific PRO instruments, five were respiratory health-related quality of life (HRQoL) instruments, six were generic or general HRQoL instruments, and six were HRQoL instruments to measure other conditions. Cough frequency, cough severity, and cough quality (cough-related symptoms and disruptions to functioning related to cough) were identified as concepts of interest to evaluate patients’ experience of the severity of RCC. Based on the literature, the most frequently reported RCC-related impacts were related to sleep, pain, and depression or anxiety.

In Stage 2, three clinical experts reported that both the frequency and the severity of coughing are troublesome for patients with RCC. Non-frequent but severe coughing may be more impactful to patients than a frequent, non-severe cough. The most common symptoms of RCC reported by the clinical experts were coughing, dyspnea, voice disorders, hoarseness, incontinence, and headaches. One of the most commonly reported impacts of RCC was embarrassment leading to stigma and social isolation. Clinicians considered self-consciousness when coughing to be an important impact, caused by concern about how others might react or whether the patient is thought of as having an illness or disease. Another important impact of RCC, mentioned by all three clinical experts, was stress-induced urinary incontinence in women. This symptom was particularly troublesome for patients, who adapt their lifestyles and take preventative action to avoid embarrassment. The clinical experts reported having experience using PROs to collect data from patients and commented that PROs were used for data collection in clinical trials, not in general clinical practice. Two clinical experts suggested using an incontinence PRO to assess the impact of this symptom in a clinical trial. Also in Stage 2 the regulatory consultant recommended that data be presented from the patient perspective on reduction of cough frequency, intensity, and disruption.

In Stage 3, the preliminary conceptual framework for the first version of the SCCD (Version 0.1) was developed to map the concepts of interest identified from Stage 1 and Stage 2 (Additional File 1: Supplemental Fig. S1). Based on the literature review and clinical expert and regulatory consultant interviews, the experiences of patients with RCC were likely to vary day to day; therefore, a 24-hour recall period and, for ease of operation, an electronic daily diary format was suggested for the SCCD. Version 0.1 of the SCCD had 11 items assessing the concepts of: cough frequency (four items), cough intensity/bothersomeness (four items), and cough-related sleep disruption (three items). Two types of response options were drafted: 11-point numeric rating scales and five-point verbal rating scales.

**Fig. S1** Preliminary conceptual framework for the SCCD Version 0.1


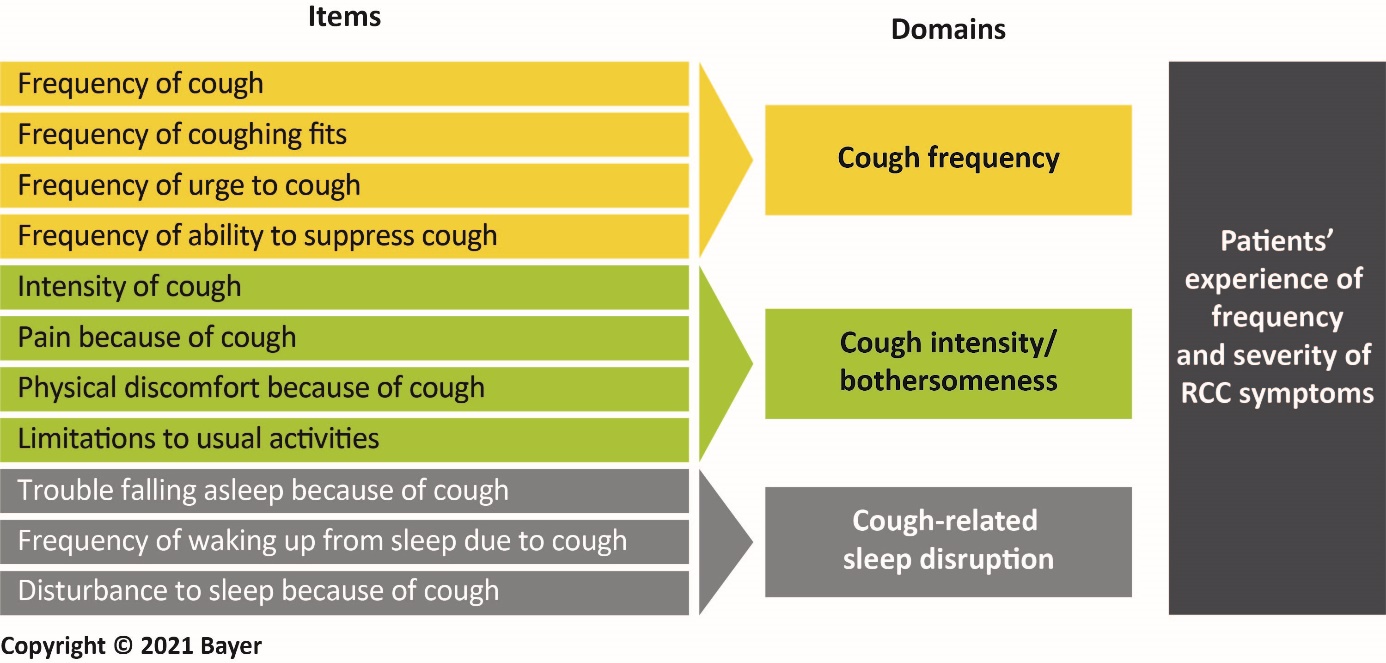


*RCC* refractory chronic cough, *SCCD* Severity of Chronic Cough Diary

**Table S1** Self-reported cough-related clinical characteristics of participants

| **Characteristics** | **Round 1 (n = 9)** | **Round 2 (n = 10)** | **Round 3 (n = 10)** | **Total (*N* = 29)** |
| --- | --- | --- | --- | --- |
| **Time since diagnosis of RCC, n (%)** | | | | |
| 6 months to <1 year ago | 1 (11) | 0 (0) | 0 (0) | 1 (3) |
| 1 year to <3 years ago | 2 (22) | 4 (40) | 1 (10) | 7 (24) |
| 3 years to <5 years ago | 2 (22) | 1 (10) | 0 (0) | 3 (10) |
| 5 years to <10 years ago | 3 (33) | 3 (30) | 1 (10) | 7 (24) |
| ≥10 years ago | 1 (11) | 2 (20) | 7 (70) | 10 (34) |
| Missing | 0 (0) | 0 (0) | 1 (10) | 1 (3) |
| **Patient-reported current severity of cough (at point of recruitment), n (%)** | | | | |
| Mild | 0 (0) | 1 (10) | 3 (30) | 4 (14) |
| Moderate | 2 (22) | 6 (60) | 5 (50) | 13 (45) |
| Severe | 5 (56) | 3 (30) | 2 (20) | 10 (34) |
| Very severe | 1 (11) | 0 (0) | 0 (0) | 1 (3) |
| Missing | 1 (11) | 0 (0) | 0 (0) | 1 (3) |
| **Patient-reported specific cough-related experiences (past 4 weeks at point of recruitment)^a^, n (%)** | | | | |
| Frequent cough | 7 (78) | 7 (70) | 3 (30) | 17 (59) |
| Intense cough | 6 (67) | 3 (30) | 1 (10) | 10 (34) |
| Coughing fits, spasms, or episodes | 8 (89) | 5 (50) | 6 (60) | 19 (66) |
| Coughing up phlegm/sputum | 5 (56) | 2 (20) | 3 (30) | 10 (34) |
| Dry cough | 7 (78) | 6 (60) | 7 (70) | 20 (69) |
| Chest discomfort | 3 (33) | 3 (30) | 3 (30) | 9 (31) |
| Abdominal discomfort | 4 (44) | 3 (30) | 5 (50) | 12 (41) |
| Rib or intercostal pain | 3 (33) | 3 (30) | 2 (20) | 8 (28) |
| **Coughing fits (past 24 hours)** | | | | |
| Number of participants experiencing coughing fits, n (%) | — | — | 8 (80) | 8 (80)^b^ |
| Mean (SD) number of coughing fits | — | — | 4 (4) | 4 (4)^b^ |
| Median [range] number of coughing fits | — | — | 3 [0–12] | 3 [0–12]^b^ |
| **Cough during usual activities (at point of recruitment), n (%)** | | | | |
| No | 0 (0) | 1 (10) | 0 (0) | 1 (3) |
| Yes, a little | 0 (0) | 3 (30) | 4 (40) | 7 (24) |
| Yes, some | 6 (67) | 5 (50) | 5 (50) | 16 (55) |
| Yes, a lot | 3 (33) | 1 (10) | 1 (10) | 5 (17) |
| Very bothersome | 3 (33) | 1 (10) | 0 (0) | 4 (14) |
| Moderately bothersome | 4 (44) | 3 (30) | 7 (70) | 14 (48) |
| Not at all bothersome | 0 (0) | 2 (20) | 1 (10) | 3 (10) |
| Missing | 2 (22) | 4 (40) | 2 (20) | 8 (28) |
| **Cough when trying to sleep (at point of recruitment), n (%)** | | | | |
| No | 1 (11) | 1 (10) | 1 (10) | 3 (10) |
| Yes, a little | 0 (0) | 1 (10) | 3 (30) | 4 (14) |
| Yes, some | 4 (44) | 6 (60) | 4 (40) | 14 (48) |
| Yes, a lot | 4 (44) | 2 (20) | 2 (20) | 8 (28) |
| Very bothersome | 3 (33) | 4 (40) | 1 (10) | 8 (28) |
| Moderately bothersome | 3 (33) | 1 (10) | 6 (60) | 10 (34) |
| Not at all bothersome | 0 (0) | 1 (10) | 1 (10) | 2 (7) |
| Missing | 3 (33) | 4 (40) | 2 (20) | 9 (31) |
| **Work missed (past 30 days) due to RCC^c^** | | | | |
| Number of participants who missed work, n (%) | 6 (67) | 5 (50) | 6 (60) | 17 (59) |
| Mean (SD) number of days of work missed | 1 (1) | 3 (3) | 1 (2) | 2 (2) |
| Median [range] number of days of work missed | 1 [0–3] | 1 [0–7] | 0 [0–6] | 0 [0–7] |
| N/A^d^, n (%) | 4 (44) | 5 (50) | 4 (40) | 13 (45) |
| **Treatments being used for cough (at point of recruitment)^a^, n (%)** | | | | |
| Doctor’s prescription | 5 (56) | 5 (50) | 8 (80) | 18 (62) |
| Over-the-counter treatment | 4 (44) | 3 (30) | 6 (60) | 13 (45) |
| Herbal remedy | 3 (33) | 1 (10) | 3 (30) | 7 (24) |
| Other^e^ | 2 (22) | 3 (30) | 2 (20) | 7 (24) |
| **Patient-reported effectiveness of their current treatment, n (%)** | | | | |
| Completely | 1 (11) | 0 (0) | 1 (10) | 2 (7) |
| Moderately | 0 (0) | 1 (10) | 4 (40) | 5 (17) |
| Somewhat | 6 (67) | 2 (20) | 3 (30) | 11 (38) |
| Slightly | 1 (11) | 5 (50) | 1 (10) | 7 (24) |
| Not at all | 1 (11) | 1 (10) | 1 (10) | 3 (10) |
| Missing | 0 (0) | 1 (10) | 0 (0) | 1 (3) |
| **Other self-reported health condition^a^, n (%)** |  |  |  |  |
| None | 2 (22) | 6 (60) | 0 (0) | 8 (28) |
| Allergic rhinitis | 1 (11) | 2 (20) | 1 (10) | 4 (14) |
| Asthma  Mild  Moderate  Severe  Missing | 5 (56)^f^  3 (33)  2 (22)  1 (11)  3 (33) | 3 (30)  2 (20)  1 (10)  0 (0)  7 (70) | 8 (80)  2 (20)  6 (60)  0 (0)  2 (20) | 16 (55)  7 (24)  9 (31)  1 (3)  12 (41) |
| Angina | 1 (11) | 1 (10) | 0 (0) | 2 (7) |
| Cancer | 1 (11) | 0 (0) | 0 (0) | 1 (3) |
| GERD  Mild  Moderate  Missing | 3 (33)  1 (11)  2 (22)  6 (67) | 2 (20)  1 (10)  1 (10)  8 (80) | 1 (10)  0 (0)  1 (10)  9 (90) | 6 (21)  2 (7)  4 (14)  23 (79) |
| Post-nasal drip syndrome | 2 (22) | 1 (10) | 2 (20) | 5 (17) |
| Other^g^ | 0 (0) | 1 (10) | 2 (20) | 3 (10) |
| **Patient-reported overall health, n (%)** | | | | |
| Very good | 2 (22) | 3 (30) | 5 (50) | 10 (34) |
| Good | 4 (44) | 5 (50) | 3 (30) | 12 (41) |
| Fair | 2 (22) | 2 (20) | 1 (10) | 5 (17) |
| Poor | 1 (11) | 0 (0) | 1 (10) | 2 (7) |

^a^Participants were permitted to select all that apply.

^b^Total *N* = 10 rather than 29 as feedback was obtained only during Round 3.

^c^Includes one participant who reported N/A and 0 days of work missed.

^d^The N/A option was provided for patients who did not work.

^e^Other treatments being used for cough included: breathing exercises (n = 1); drinking water (n = 1); face steams (n = 1); meditation/mindfulness (n = 1); sleep/relaxation
(n = 1); Vicks VapoRub (n = 1); breathing slowly and reducing stress (n = 1).

^f^For Round 1, one participant selected both mild and moderate.

^g^Other self-reported health conditions included: seasonal allergies (n = 1); hypothyroidism
(n = 1); vertigo, ocular rosacea, oral lichen planus, incontinence (n = 1).

*GERD* gastroesophageal reflux disease, *N/A* not applicable, *RCC* refractory chronic cough, *SD* standard deviation

**Table S2** Item tracking matrix showing changes to SCCD concept coverage, instructions, recall period, and response options

|  | **SCCD Version 0.1** | **SCCD Version 0.2** | **SCCD Version 0.3** | **SCCD Version 1.0** |
| --- | --- | --- | --- | --- |
| Concept coverage | 11 items | 15 items  Items added on urinary incontinence, ability to do strenuous physical activities, impact on social interactions, and difficulty staying awake the next day due to coughing the night before, based on participants’ and clinical expert feedback on the SCCD Version 0.1 | 16 items  An item on breathlessness was added as "breathlessness" was a missing concept in the transcript evaluation by the instrument development team | 14 items  Two sleep items—frequency of waking from sleep and the impact of sleep disturbance on the ability to stay awake the next day—were removed due to participants reporting redundancy between some sleep items |
| Instructions | — | Following suggestions from two participants (22%) in Round 1, the statements “in the daytime and nighttime” and “answer all questions” were removed to simplify the instructions | After Round 2, the phrase “select only one option for each question” was added based on recommendations from the translatability team | No further changes were made following Round 3 |
| Recall period | Two participants based their responses on the time of their interview (morning), rather than if they were to complete the diary at night. A third participant reported that 24 hours did not capture seasonal variation in symptoms. None of these participants had difficulty interpreting "the past 24 hours." Additionally, one participant specifically reported a preference for completing items on a daily basis | No changes made  Three participants seemed to apply a different 24-hour recall (not based on interview time to the same time on the previous day) | No changes made  Three participants seemed to apply a different 24-hour recall (not based on interview time to the same time on previous day) | No changes made |
| Response options | 11-point NRS | The 11-point NRS was changed to a five-point VRS after Round 1 based on participants’ responses to frequency items in order to avoid confusion with the exact number of cough/symptoms experienced. For items assessing symptom severity the VRS was maintained | In Round 2, some participants queried the difference between the “a lot” and “extreme” response anchors; as such, the “extreme” option was removed. A “no sleep disturbance” response option was also added to the disturbance of sleep due to cough item in response to participant feedback in Round 2. After Round 2, a “no cough” option was added to items assessing the impact of cough in the previous 24 hours (e.g., cough control, cough-related pain). The term “constantly” in the VRS was also amended to “almost constantly,” as participant feedback and the instrument development team deemed constant coughing not to be likely or feasible | A “not applicable” response option for the disruptions to strenuous physical activities item was added based on feedback from participants in Round 2 and Round 3 who responded that they had not engaged in strenuous physical activities in the past 24 hours |

*NRS* numeric rating scale, *SCCD* Severity of Chronic Cough Diary, *VRS* verbal rating scale

**Table S3** Participants’ SCCD scores

| **SCCD score** | **Round 1**  NRS^a^, n = 9 | **Round 2**  VRS, n = 10 | **Round 3**  VRS, n = 10 |
| --- | --- | --- | --- |
| **Cough symptoms** | | | |
| Frequency of cough | Mean (SD): 6.8 (3.4)  Median [range]: 7 [0–10]  Mode: 10 | Never: 0 (0)  Rarely: 1 (10)  Sometimes: 4 (40)  Frequently: 5 (50)  Constantly: 0 (0) | Never: 0 (0)  Rarely: 1 (10)  Sometimes: 6 (60)  Frequently: 2 (20)  Almost constantly: 1 (10) |
| Severity of cough | Mean (SD): 5.4 (3.0)  Median [range]: 5 [0–10]  Mode: 5 | No cough: 1 (10)  Mild: 4 (40)  Moderate: 4 (40)  Severe: 1 (10)  Very severe: 0 (0) | No cough: 1 (10)  Mild: 3 (30)  Moderate: 4 (40)  Severe: 2 (20)  Very severe: 0 (0) |
| Coughing fits | Mean (SD): 4.4 (3.1)  Median [range]: 5 [0–9]  Mode: 7 | Never: 5 (50)  Rarely: 2 (20)  Sometimes: 3 (30)  Frequently: 0 (0)  Constantly: 0 (0) | Never: 2 (20)  Rarely: 0 (0)  Sometimes: 4 (40)  Frequently: 3 (30)  Almost constantly: 1 (10) |
| Urge to cough | Mean (SD): 6.0 (3.0)  Median [range]: 7 [0–10]  Mode: 7 | Never: 1 (10)  Rarely: 1 (10)  Sometimes: 2 (20)  Frequently: 6 (60)  Constantly: 0 (0) | Never: 0 (0)  Rarely: 1 (10)  Sometimes: 5 (50)  Frequently: 2 (20)  Almost constantly: 2 (20) |
| Ability to control cough | Mean (SD): 4.4 (3.2)  Median [range]: 4 [0–10]  Mode: 4 | Never: 2 (20)  Rarely: 0 (0)  Sometimes: 6 (60)  Frequently: 1 (10)  Always: 1 (10) | No cough: 0 (0)  Never: 0 (0)  Rarely: 3 (30)  Sometimes: 5 (50)  Frequently: 2 (20)  Almost constantly: 0 (0) |
| **Symptoms related to cough** | | | |
| Pain due to cough | Mean (SD): 2.6 (2.9)  Median [range]: 2 [0–8]  Mode: 0 | None: 5 (50)  A little: 2 (20)  Some: 3 (30)  A lot: 0 (0)  Extreme: 0 (0) | No cough: 0 (0)  No pain: 4 (40)  Mild: 1 (10)  Moderate: 4 (40)  Severe: 1 (10)  Very severe: 0 (0) |
| Physical discomfort due to cough | Mean (SD): 4.2 (3.2)  Median [range]: 4 [0–8]  Mode: 8 | None: 3 (30)  A little: 2 (20)  Some: 4 (40)  A lot: 1 (10)  Extreme: 0 (0) | No cough: 1 (10)  No discomfort: 2 (20)  Mild: 3 (30)  Moderate: 3 (30)  Severe: 0 (0)  Very severe: 1 (10) |
| Breathlessness due to cough | N/A | N/A | No cough: 1 (10)  No breathlessness: 2 (20)  Mild: 4 (40)  Moderate: 2 (20)  Severe: 1 (10)  Very severe: 0 (0) |
| Leaking urine due to cough | N/A | Never: 8 (80)  Rarely: 1 (10)  Sometimes: 0 (0)  Frequently: 1 (10)  Every time: 0 (0) | No cough: 1 (10)  Never: 3 (30)  Rarely: 1 (10)  Sometimes: 2 (20)  Frequently: 2 (20)  Every time: 1 (10) |
| **Disruption to activities due to cough** | | | |
| Disruptions to usual household activities | Mean (SD): 4.7 (3.4)  Median [range]: 4 [0–10]  Mode: 4 | Not at all: 4 (40)  A little: 4 (40)  Some: 1 (10)  A lot: 1 (10)  Could not do these activities: 0 (0) | No cough: 0 (0)  Not at all: 4 (40)  A little: 2 (20)  Some: 3 (30)  A lot: 1 (10)  Could not do these activities: 0 (0) |
| Disruptions to strenuous physical activities | N/A | Not at all: 5 (50)  A little: 2 (20)  Some: 0 (0)  A lot: 3 (30)  Could not do these activities: 0 (0) | No cough: 0 (0)  Not at all: 5 (50)  A little: 0 (0)  Some: 3 (30)  A lot: 1 (10)  Could not do these activities: 1 (10) |
| Disruptions to social interaction | N/A | Not at all: 3 (30)  A little: 2 (20)  Some: 3 (30)  A lot: 2 (20)  Could not do these activities: 0 (0) | No cough: 0 (0)  Not at all: 3 (30)  A little: 3 (30)  Some: 3 (30)  A lot: 1 (10)  Could not do these activities: 0 (0) |
| **Disruption to sleep due to cough** | | | |
| Difficulty staying awake during the day due to coughing the night before | N/A | None: 6 (60)  A little: 2 (20)  Some: 1 (10)  A lot: 1 (10)  Extreme: 0 (0) | No cough: 0 (0)  No sleep disturbance: 2 (20)  Not at all: 2 (20)  A little: 3 (30)  Some: 2 (20)  A lot: 1 (10) |
| Difficulty falling asleep | Mean (SD): 4.0 (3.7)  Median [range]: 5 [0–10]  Mode: 0 | None: 4 (40)  A little: 2 (20)  Some: 4 (40)  A lot: 0 (0)  Extreme: 0 (0) | No cough: 1 (10)  Not at all: 2 (20)  A little: 2 (20)  Some: 3 (30)  A lot: 1 (10)  Could not sleep: 1 (10) |
| Disturbance of sleep | Mean (SD): 3.0 (3.7)  Median [range]: 2 [0–10]  Mode: 0 | Not at all: 4 (40)  Slightly: 4 (40)  Moderately: 1 (10)  Severely: 1 (10)  Could not sleep: 0 (0) | No cough: 1 (10)  Not at all: 3 (30)  A little: 0 (0)  Some: 4 (40)  A lot: 1 (10)  Could not sleep: 1 (10) |
| Waking up from sleep | Mean (SD): 2.9 (3.5)  Median [range]: 2 [0–9]  Mode: 0 | Never: 5 (50)  Rarely: 1 (10)  Sometimes: 3 (30)  Frequently: 1 (10)  Constantly: 0 (0) | No cough: 1 (10)  Not at all: 4 (40)  A little: 0 (0)  Some: 3 (30)  A lot: 1 (10)  Could not sleep: 1 (10) |

Data are n (%) unless otherwise stated. ^a^11-point NRS range 0–11, where a higher score indicates a worse cough experience.

*N/A* not applicable, *NRS* numeric rating scale, *SCCD* Severity of Chronic Cough Diary, *SD* standard deviation, *VRS* verbal rating scale

**Supplementary references**

1. Patrick DL, Burke LB, Gwaltney CJ, Leidy NK, Martin ML, Molsen E et al (2011) Content validity–establishing and reporting the evidence in newly developed patient-reported outcomes (PRO) instruments for medical product evaluation: ISPOR PRO good research practices task force report: part 1–eliciting concepts for a new PRO instrument. Value Health 14(8):967–977.
2. Patrick DL, Burke LB, Gwaltney CJ, Leidy NK, Martin ML, Molsen E et al (2011) Content validity–establishing and reporting the evidence in newly developed patient-reported outcomes (PRO) instruments for medical product evaluation: ISPOR PRO good research practices task force report: part 2–assessing respondent understanding. Value Health 14(8):978–988.
