# Supplementary material for: The Severity of Chronic Cough Diary (SCCD): development and content validation of a novel patient-reported outcome instrument for evaluating the symptom experience of chronic cough
Source: J Patient Rep Outcomes. 2023 Jul 10;7:65. doi: 10.1186/s41687-023-00605-8 (PMC10333155; doi:10.1186/s41687-023-00605-8)
Supplement: Supplementary file 2 — Supplementary Material 2 [file 41687_2023_605_MOESM2_ESM.docx]

## Abstract

**Background**: Refractory chronic cough (RCC), a cough lasting longer than 8 weeks with an unexplained underlying etiology and unresponsive to conventional treatment, can have substantial effects on patients’ quality of life. For assessment of the efficacy of antitussive medication in clinical trials in RCC, patient-reported outcome (PRO) instruments should be fit for purpose with appropriate content validity. Here we describe the qualitative testing of a newly developed PRO instrument: the Severity of Chronic Cough Diary (SCCD).

**Methods**: The SCCD was developed to assess patients’ symptom experience of cough in patients with RCC. A preliminary version was tested and refined based on an iterative process in a qualitative study. In total, three rounds of interviews were conducted with adult participants diagnosed with RCC in the USA (n = 19) and UK (n = 10). Rounds 1–3 consisted of hybrid concept elicitation (CE) interviews and cognitive interviews (CIs), with Round 3 also including interviews in a subset of participants (n = 5) about the usability of the SCCD as administered on an electronic handheld device.

**Results**: The CE interviews identified concepts important to patients’ experiences related to RCC that were broadly in line with the concepts in the preliminary version of the SCCD. Participants provided positive feedback on the draft SCCD across all CI rounds, reporting the instrument to be relevant and straightforward to complete, and containing a comprehensive set of concepts to evaluate their symptom experience of RCC. Participants demonstrated a good understanding of proposed item wording, response options, and the 24-hour recall period, and thought completion of the SCCD on the electronic device was easy. Following revisions based on results from each interview round, the SCCD at the end of this qualitative research study had 14 items assessing the concepts of: cough symptoms (five items), symptoms related to cough (four items), disruption to activities due to cough (three items), and disruption to sleep due to cough (two items).

**Conclusions**: The results of this study provide qualitative evidence supporting the content validity of the SCCD as a PRO instrument for evaluating outcomes of therapies for RCC in clinical trials.

**Keywords:** Refractory chronic cough, RCC, Refractory unexplained chronic cough, Severity of Chronic Cough Diary, Content validity, Patient-reported outcome, PRO, Qualitative, Diary

## Introduction

Refractory chronic cough (RCC)—defined here as a cough lasting longer than 8 weeks with an unexplained underlying etiology and which is unresponsive to conventional treatment [1]—is a significant cause of morbidity, affecting around 10% of adults globally [2]. In addition to physical effects, RCC can have a substantial impact on patients’ health-related quality of life (HRQoL) [3, 4]. Primary endpoints in RCC clinical trials typically evaluate change in the frequency of cough using objective monitoring devices [5-7]. Regulatory agencies such as the European Medicines Agency and the US Food and Drug Administration (FDA) emphasize the importance of patient perspectives in treatment evaluation [8-11]. As a prerequisite, fit-for-purpose patient-reported outcome (PRO) instruments have to be used to assess the frequency and severity of cough from the patient perspective for evaluating outcomes of novel targeted antitussive agents for RCC, such as purinergic P2X3 receptor antagonists [12]. For the development of these PRO instruments, qualitative research is an essential step to ensure the instrument captures and evaluates important aspects of patients’ experiences in clinical trials [8-11, 13].

PRO instruments, such as the Leicester Cough Questionnaire [14] and the Cough-Specific Quality of Life Questionnaire [15], have been used widely to assess patients’ experiences of cough and its impact on HRQoL for endpoints in RCC clinical trials [5-7, 16-18]. The Cough Severity Diary (CSD) was later developed to better quantify cough severity (frequency, intensity, and disruption related to cough) in order to evaluate the effects of new therapies [19]. The CSD has been used in clinical trials of gefapixant in RCC [6, 7]. However, as the CSD was not available for external commercial licensing, a new PRO instrument was required for investigating new therapeutic options for RCC, such as in clinical trials of the P2X3 antagonist eliapixant [20].

This article describes the development of a new PRO instrument, the Severity of Chronic Cough Diary (SCCD), that is designed to assess treatment efficacy in RCC clinical trials. We report the qualitative testing phase of SCCD development, with a brief overview of the preliminary development phases.

## Methods

### Overview of SCCD development

The SCCD was developed using an iterative process (Fig. 1). In line with FDA guidance on PRO development [8], a preliminary conceptual framework (Additional File 1: Supplemental Fig. S1) for measuring patient-reported frequency and severity of cough and related symptoms in a clinical trial setting was developed based on the results of a targeted literature review (Stage 1) and interviews with three clinical experts and one regulatory consultant (Stage 2) conducted in 2018. This preliminary conceptual framework guided the development of the first draft of the SCCD Version 0.1 (Stage 3). Stages 1–3 are briefly described in the Supplementary Information.

Revisions to the SCCD leading to Versions 0.2, 0.3, and 1.0 and the updated conceptual framework were based on participant feedback from the qualitative research study which is described here (Stage 4). Best practice methods were followed to ensure that robust evidence was collected to support the content validity of the SCCD [8, 21, 22].

### Qualitative research study to assess the content validity of the SCCD

#### Study design

Stage 4 was a cross-sectional, non-interventional, qualitative interview study involving individual, in-person, and telephone interviews with adults with RCC in the USA and UK. This study was approved by a central Institutional Review Board in the USA (Ethical & Independent Institutional Review Board [Study 19056-01]). Approval of this study was not required by the UK Health Research Authority because participants were not recruited via the National Health Service (NHS). All study participants provided written informed consent.

The three rounds of interviews were conducted with a planned total cohort of approximately 30 participants from the USA and UK (Fig. 1). Rounds 1 and 2 consisted of hybrid concept elicitation (CE) interviews and cognitive interviews (CIs) conducted via telephone and lasted approximately 90 min. For Round 3, half the participants took part in telephone interviews (60 min) and the other half took part in in-person interviews (75 min). The 60-min telephone interviews consisted of hybrid CE interviews and CIs. The 75-min in-person interviews utilized an electronic version of the SCCD and consisted of hybrid CE interviews and CIs (60 min), and usability testing (15 min). For all Round 3 interviews, the CE section could be shortened as concept saturation for cough severity and frequency concepts had already been achieved in Rounds 1 and 2.

Interviews were conducted by researchers experienced in qualitative interviewing techniques. The SCCD was completed by the study participants and all interviews followed a semi-structured interview guide and were audio recorded with participant permission. After each interview round, revisions to the SCCD were made based on participant feedback and input from the instrument development team (AH, AS, CH, HK, MdlOA). These revisions were documented in an item tracking matrix and were further tested in subsequent interview rounds. Additional feedback on SCCD Versions 0.1 and 0.3 was provided by one of the clinicians who previously took part in the Stage 2 interviews. The clinician had expertise in managing patients with RCC and evaluating the outcomes of interventions, and this feedback informed the clinical relevance of the concepts included in the first draft of the SCCD before the patient interviews were initiated. Also, a translatability assessment of the SCCD Version 0.2, including evaluation for neutrality or “universal” English so that the instrument would be appropriate for use across different English-speaking countries, was conducted by a translation expert. The SCCD Version 0.3 was adapted for administration on an electronic handheld device in a subset of participants. The combination of study participant and clinical expert feedback on the SCCD Version 0.3 led to the finalization of the SCCD Version 1.0.

#### Participant recruitment and eligibility

Participants were recruited to one of three rounds of interviews via convenience sampling that used a recruitment vendor’s proprietary database, social media, healthcare professional referrals (USA only), and patient associations. As this was a qualitative study, sample size determination was based on concept saturation with no formal calculation possible [11]; however, 85% of symptom concepts in qualitative research studies have been shown to emerge after 10 interviews [23].

Inclusion and exclusion criteria for the study were chosen to allow for a study population comparable to clinical trials in patients with RCC. Eligible participants were adults (aged ≥18 years) residing in the USA or UK with a self-reported diagnosis/history of chronic cough, defined as a cough lasting for ≥8 weeks that had persisted ≥1 year as refractory or idiopathic. Full inclusion and exclusion criteria can be found in the Supplementary methods. Physician confirmation of RCC diagnosis was not an eligibility requirement in general. However, some USA study participants shared physician confirmation of diagnosis after screening.

#### Detailed description of the interview procedures

*Concept elicitation* The objective of the CE part of the interviews was to investigate whether concepts in the SCCD Versions 0.1–0.3 were relevant and would comprehensively capture the experiences of patients with RCC. Participants were first asked open-ended questions about their experiences of RCC in terms of its symptoms and impacts. Participants were then asked to describe their cough on a typical day, a good day, and a bad day, and to provide a comprehensive description of their cough symptoms, symptoms related to cough, disruptions to daily life due to impacts of coughing, ability to control their cough, physical impacts of coughing, and their current experience of treatment and its effectiveness. The concepts and experiences spontaneously reported by patients were recorded. Participants were then probed by interviewers to determine whether concepts which had not been mentioned spontaneously by the participant (for example, physical discomfort due to cough was spontaneously mentioned by 4 [14%] participants across the interview rounds), but which were included in the preliminary conceptual framework, were relevant to their experience.

*Cognitive interviewing* The primary objective of the CIs was to evaluate participants’ understanding of the item stems, recall period, and response options of the respective draft versions of the SCCD (Versions 0.1–0.3). In particular, participants were asked to complete the SCCD, to describe their initial impressions of the diary, and provide feedback on each item stem and the response options. This was to ascertain the participants’ understanding and the relevance of each item to their experience of cough, and also to provide any suggestions for changes. The suitability and relevance of the 11-point numeric rating scale (NRS) and the five-point verbal rating scale (VRS) response options were also evaluated.

*Usability testing* Once the suitability of the wording of the SCCD had been evaluated by participants in Round 1 and Round 2 interviews, usability testing was conducted in Round 3 with participants who were administered the SCCD loaded on an electronic handheld device (Bluebird Android handheld device; eResearch Technology, USA). Following a brief explanation of how the electronic diary was to be used during the study, participants were asked to complete the SCCD on the electronic handheld device and provide feedback on its usability.

#### Data analysis

Descriptive statistics of sociodemographic data and clinical characteristics were calculated using SAS 9.4 software. A codebook for both CE and CI qualitative data was developed for each round of interviews based on the interview discussion guide. After coding of the first transcript, the coding framework was revised as necessary, and the remaining transcripts were then coded accordingly using ATLAS.ti Version 8.0.

Content analysis of RCC symptoms and impacts reported during the CE part of the interviews was conducted using ATLAS.ti Version 8.0 to identify key concepts of interest (COI). Concept saturation was determined using a deductive approach and documented using a saturation grid, iteratively evaluated over the three rounds of interviews. Concept saturation was evaluated by identifying the first instance a concept was mentioned within the chronologic order of data collection, and determined to be achieved if no new concepts were reported over two or more interviews.

For analyses of the CIs, the coding framework reported the participants’ feedback on the comprehension, relevance, and acceptability of items in the SCCD.

## Results of the qualitative research study

### Participant demographics

In total, 29 participants from the USA (n = 19) and UK (n = 10) were interviewed for the Stage 4 qualitative research study between May 14, 2019 and November 12, 2019. All interviews in Round 1 (n = 9) and Round 2 (n = 10) were conducted by telephone. In Round 3 (n = 10), five interviews using the paper-based version of the SCCD Version 0.3 were conducted by telephone and five interviews using the electronic version of the SCCD Version 0.3 were conducted in person. The self-reported sociodemographic characteristics of the participants are shown in Table 1.

**TABLE 1 TO BE PLACED HERE**

### Participant clinical characteristics

Self-reported cough-related clinical characteristics of the participants are shown in Additional File 1: Supplemental Table S1. Participants were most commonly diagnosed with RCC at least 10 years before enrollment (n = 10; 34%) and most participants rated their cough as “moderate” (n = 13; 45%) or “severe” (n = 10; 34%).

### Concept elicitation results

#### Concepts of interest reported during the concept elicitation part of the interviews

COI related to patients’ symptom experience of RCC were spontaneously elicited during patient descriptions of typical days, good days, and bad days with RCC. The frequencies of the spontaneously described concepts mentioned during the three interview rounds and example participant quotes are shown in Table 2. Concept saturation for disruptions due to cough and cough symptoms/symptoms related to cough was achieved within Round 1 and Round 2 of CE interviews, respectively (Table 2).

**TABLE 2 TO BE PLACED HERE**

#### Cough symptoms

Experiences related to the ability to control cough was the most common concept relating to cough symptoms. This concept was included in the preliminary version of the SCCD as an “ability to suppress cough,” but participants referred to this more as an “ability or inability to control” their cough. The vast majority of participants described their experience with cough in terms of frequency, ranging daily from “always,” “constantly,” “on and off,” “200 times,” and “a couple of times an hour” to “the whole day.” Accounts of the severity of cough were based on characteristics such as “bellowing,” “loudness,” and the “longevity” of cough and the “pain,” “choking feeling,” and “throwing up” associated with the constant coughing or cough episodes. Participants also provided clear descriptions of repeated coughing, most commonly referred to as “coughing fits” or “coughing spells.” Coughing fits were reported as lasting “20 to 30 seconds,” “10 to 15 mins,” or “20 mins,” or as happening “in bursts” or “all day,” and could be triggered by air temperature or quality. The urge to cough was described by participants as “something stuck in your throat,” a “tickle,” a “dryness,” an “irritation,” or a “lump” in the throat that would result in coughing if not remedied.

#### Symptoms related to cough

In total, eight participants experienced pain in their neck (n = 1), throat (n = 2), lungs (n = 1), chest (n = 1), stomach/belly/side (n = 3), muscles (n = 1), shoulder (n = 1), lower back (n = 2), and ribs (n = 1) as a result of coughing, as well as headaches (n = 2). Pain was associated with the level of intensity of cough occurring during “bad days” or a “coughing fit.” Additionally, some participants reported symptoms of physical discomfort related to coughing as “feeling drained,” having a sore throat, feeling “uncomfortable,” or having to sleep upright to avoid coughing or vomiting due to the coughing. Breathlessness due to cough was described as a “strangling” or “choking” feeling and as not being able to “catch your breath” or “not being able to breathe.”

In total, five participants described experiencing leaking urine (incontinence) due to cough. All five participants were women (22% of the women in the study), who reported that their weakened bladder muscles were the result of constant coughing. Although this concept was reported by a limited number of participants, incontinence was the most common symptom related to cough reported by all three clinical experts interviewed during Stage 2 of SCCD development. This was confirmed by the clinical expert during Stage 4 of SCCD development, and was therefore deemed important and clinically relevant to maintain in the conceptual framework.

#### Disruption to activities due to cough

Disruptions to daily activity due to cough were described by over half of the participants . This concept was included in the preliminary version of the SCCD as “usual activities,” but participants had variable interpretations of this wording. Participants distinguished between the disruptions to household activities and disruptions to strenuous activities; strenuous activities due to physical exertion from coughing included activities such as walking, exercise, and playing sports.

#### Emotional impacts due to cough

As emotional impacts were multidimensional and encompassed several psychological, emotional, and mood concepts, saturation was not reached at the symptom impact level (e.g., feeling sad, embarrassed, anxious, etc.) but only at the domain level. Given the context-specific nature of the emotional impacts, it was not possible to create an item that was clearly worded and still relevant to the majority of participants with RCC.

#### Disruption to sleep due to cough

Waking up from sleep due to coughing was reported by around three-quarters of participants, whereby a “sudden coughing fit” could “startle” them and wake them up. Disturbance of sleep due to coughing was described as having difficulty falling asleep and not being able to stay asleep, get back to sleep, or sleep soundly. Impacts on daytime performance resulting from sleeplessness from coughing the night before included: not going to work, being “not fully able to participate,” or having to “cut short” work due to difficulties with “focus” and feeling “uncoordinated” and “fatigued” the next day.

#### Support for recall period and response scale

Participants reported that their RCC could vary from day to day, supporting the 24-hour recall period for measuring symptom experiences of cough severity and the concept of a daily diary measurement. RCC on a “typical day” ranged from a predictable pattern of cough throughout the day to a changeable pattern of cough depending on the time of day. Participants described a “good day” and a “bad day” of cough in terms of the frequency of cough symptoms and their perception of severity.

Participants’ feedback on cough symptoms such as cough severity, pain, and discomfort supported the use of a severity scale, while cough frequency, urge to cough, and difficulty controlling cough appeared to be better measured with a frequency scale.

### Cognitive interview results

#### Concept coverage and revisions

Across all interview rounds, participants provided positive feedback on the SCCD in general and found the instrument to be relevant, with a comprehensive set of concepts to evaluate the symptom experience of RCC. Further information can be found in Additional File 1: Supplemental Table S2.

After Round 1, the number of items in the SCCD was increased from 11 in Version 0.1 to 15 in Version 0.2; with items on urinary incontinence, ability to do strenuous physical activities, impact on social interactions, and difficulty staying awake the next day due to coughing the night before were added for testing in Round 2 (SCCD Version 0.2). After Round 2, an item on breathlessness was added to the SCCD Version 0.3 (16 items) for evaluation in Round 3. After Round 3, two sleep items—frequency of waking from sleep and the impact of sleep disturbance on the ability to stay awake the next day—were removed, leaving 14 items in the final SCCD Version 1.0. The revised conceptual framework of the SCCD is shown in Fig. 2.

#### Understanding and interpretation of the SCCD instructions, recall period, and response options

*Instructions and recall period* Throughout all three interview rounds, all participants (n = 29; 100%) found the instructions clear and easy to understand. The 24-hour recall period was clearly understood, with participants generally thinking back to the same time yesterday. Further information can be found in Additional File 1: Supplemental Table S2.

*Response options* The 11-point NRS of the SCCD Version 0.1 was changed to a five-point VRS after Round 1 based on participant feedback. Both NRS and VRS options were tested for relevance in Round 2, with the VRS format generally being preferred over the NRS. After Round 2 a “no cough” option was added to items assessing the impact of cough in the previous 24 hours (e.g., cough control, cough-related pain). Further information can be found in Additional File 1: Supplemental Table S2.

#### Understanding and interpretation of individual items

An overview of participants’ understanding of individual items and revisions to the SCCD based on CI feedback is shown in Table 3. Across the three rounds of CIs, participants demonstrated clear understanding of the item stems and interpreted them in a consistent manner. Participants’ symptom experience of RCC as measured by SCCD scores during each interview round is shown in Additional File 1: Supplemental Table S3.

**TABLE 3 TO BE PLACED HERE**

#### Electronic version of the SCCD usability study results

In Round 3, all five participants (female, n = 4; age range 31–62 years) who completed the SCCD on an electronic handheld device were asked about their user experience. None of these five participants had previously used an electronic PRO device, and one participant had not previously used a smartphone. All five participants (100%) thought the electronic handheld device was simple and easy to use and had no difficulty reading the text on the screen.

## Discussion

In line with FDA guidance on PRO development [8], the SCCD was developed to evaluate patients’ symptom experiences of RCC, with the objective of assessing treatment efficacy in clinical trials. The de-novo development of the SCCD was necessary, as an alternative instrument was not available for external commercial licensing at the time of the current study. Overall, the concepts that emerged from the literature review and interview feedback from three clinical experts were found to be broadly relevant to the study cohort of 29 adults with RCC. Moreover, results from the qualitative research study, consisting of CE interviews, CIs, and usability testing of the electronic handheld device, as well as feedback from a clinical expert, supported the content validity of the SCCD among patients with RCC.

After Round 1 of CE interviews, the SCCD Version 0.1 was confirmed to evaluate concepts that are important to patients with RCC. Based on the additional symptoms related to cough (cough-induced incontinence) and its impacts (on strenuous physical activities, social interactions, and difficulty staying awake during the next day) that emerged from Round 1, items were developed and tested in subsequent rounds of interviews. One item (ability to “suppress” cough) was revised (ability to “control” cough) to better reflect participants’ experiences related to RCC. CIs to assess the SCCD suggested that the instrument was easy to complete and understand, with most participants not finding any aspect of the SCCD (i.e., instructions, item stems, response scale, and recall period) confusing. Those participants asked found the electronic assessment method of the SCCD easy to use.

The SCCD at the end of the qualitative research study (Version 1.0) included 14 items for evaluating key dimensions of participants’ experiences related to RCC: five items about cough symptoms, four items about symptoms related to cough, three items about the disruption to activities due to cough, and two items that capture the disruption to sleep due to cough. Although only five women out of 23 (22%) raised the issue of urinary incontinence during the study, incontinence was noted by clinicians participating in the development of the SCCD to be clinically relevant and therefore expected on an RCC questionnaire. However, based on participant feedback during this study it may be valuable to evaluate the inclusion of a “not applicable” response option, particularly for men, during further testing.

Compared with the more recently developed SCCD and CSD, older PROs such as the Leicester Cough Questionnaire and the Cough-Specific Quality of Life Questionnaire were developed before current best practice guidance [24] and were not designed to directly assess cough severity [14, 15, 19]. A benefit of the SCCD is that it includes items assessing symptom experience of cough, including cough severity, which were identified as important concepts of interest by qualitative research in the current study. Respective measures assessing these important concepts of interest might need to be added to alternative appropriate patient-focused measurement strategies. Compared with the 14 items and four domains of the SCCD Version 1.0, the CSD has seven items and three domains: three items assessing cough frequency, two items assessing cough intensity, and two items assessing the disruption due to cough [19]. The addition of concepts in the SCCD which patients felt were important to understand their experiences of RCC, such as cough-induced incontinence and impacts on sleep and social interactions, may be beneficial for capturing more information when evaluating the efficacy of interventions in clinical trials. The response options of the two diaries also differ, with the CSD using an 11-point NRS [19] compared with the VRS of the SCCD as a result of the qualitative research in this study.

The main strengths of the study are that the SCCD was developed using current best practice standards with robust and state-of-the-art methodology and reflects up-to-date understanding of RCC [21, 22, 24]. An additional strength of the study is that the patient population was broadly similar to patients recruited to recent clinical trials of antitussive therapies (e.g., enrollment of patients with RCC and exclusion of smokers and patients with significant lung disease or recent respiratory tract infections) [5-7, 20]. However, some participants reported low scores on the SCCD in the past 24 hours. This could likely be explained by the reported day-to-day variability of cough and the nature of the interview participation; participants may have scheduled their interview on a day when they were not experiencing cough exacerbation, as noted by several participants who requested to reschedule their interview. Another limitation of the study is that the sample of participants did not represent individuals at lower education levels as in the current study most participants in the USA had at least some college education and most UK participants had a university degree. Additional testing in individuals with lower education levels is suggested. As physician confirmation of RCC diagnosis was not an eligibility requirement for this study, exit interviews with participants from phase 2 trials with clinically confirmed RCC should be considered for the future.

Psychometric analyses of the SCCD Version 1.0 are planned to provide evidence of construct validity, reliability, sensitivity to change, and interpretability of SCCD scores. The conceptual framework might be revised as the SCCD is tested in future research to make sure that the finalized versions of both the conceptual framework and the SCCD are accurately representing patients’ experiences of the frequency and severity of cough in the context of clinical trials.

## Conclusions

The ability to assess the frequency and severity of RCC and its related symptoms together with the day-to-day disruptions that RCC imposes on patients’ lives is important for evaluating the efficacy of interventions for RCC in clinical trials. In this article, we described the development and refinement of a new PRO instrument—the SCCD—based on qualitative research with patients with RCC and continuous expert input. The results of the study provide evidence supporting the content validity of the SCCD for evaluating outcomes of therapies for RCC.

## References

1. Morice AH, Millqvist E, Bieksiene K, Birring SS, Dicpinigaitis P, Domingo Ribas C et al (2020) ERS guidelines on the diagnosis and treatment of chronic cough in adults and children. Eur Respir J 55(1):1901136.

2. Song WJ, Chang YS, Faruqi S, Kim JY, Kang MG, Kim S et al (2015) The global epidemiology of chronic cough in adults: a systematic review and meta-analysis. Eur Respir J 45(5):1479–1481.

3. Morice A, Dicpinigaitis P, McGarvey L, Birring SS (2021) Chronic cough: new insights and future prospects. Eur Respir Rev 30(162):210127.

4. Dicpinigaitis PV, Birring SS, Blaiss M, McGarvey LP, Morice AH, Pavord ID et al (2023) Demographic, clinical, and patient-reported outcome data from 2 global, phase 3 trials of chronic cough. Ann Allergy Asthma Immunol 130(1):60–66.

5. Abdulqawi R, Dockry R, Holt K, Layton G, McCarthy BG, Ford AP et al (2015) P2X3 receptor antagonist (AF-219) in refractory chronic cough: a randomised, double-blind, placebo-controlled phase 2 study. Lancet 385(9974):1198–1205.

6. Muccino DR, Morice AH, Birring SS, Dicpinigaitis PV, Pavord ID, Assaid C et al (2020) Design and rationale of two phase 3 randomised controlled trials (COUGH-1 and COUGH-2) of gefapixant, a P2X3 receptor antagonist, in refractory or unexplained chronic cough. ERJ Open Res 6(4):00284–02020.

7. Smith JA, Kitt MM, Morice AH, Birring SS, McGarvey LP, Sher MR et al (2020) Gefapixant, a P2X3 receptor antagonist, for the treatment of refractory or unexplained chronic cough: a randomised, double-blind, controlled, parallel-group, phase 2b trial. Lancet Respir Med 8(8):775–785.

8. US Food Drug Administration (FDA) (2009) Patient-reported outcome measures: use in medical product development to support labeling claims—guidance for industry. <https://www.fda.gov/media/77832/download>. Accessed Oct 27, 2021.

9. European Medicines Agency (EMA) (2005) Regulatory guidance for the use of Health-related Quality of Life (HRQL) measures in the evaluation of medicinal products. <https://www.ema.europa.eu/en/documents/scientific-guideline/reflection-paper-regulatory-guidance-use-healthrelated-quality-life-hrql-measures-evaluation_en.pdf>. Accessed Oct 27, 2021.

10. US Food Drug Administration (FDA) (2019) Patient-focused drug development: methods to identify what is impotant to patients—guidance for industry, food and drug administration staff, and other stakeholders [draft guidance]. <https://www.fda.gov/media/131230/download>. Accessed Oct 27, 2021.

11. US Food Drug Administration (FDA) (2020) Patient-focused drug development: collecting comprehensive and representative input—guidance for industry, food and drug administration staff, and other stakeholders. <https://www.fda.gov/media/139088/download>. Accessed Oct 27, 2021.

12. Song WJ, Chung KF (2020) Pharmacotherapeutic options for chronic refractory cough. Expert Opin Pharmacother 21(11):1345–1358.

13. US Food Drug Administration (FDA) (2022) Patient-focused drug development: Selecting, developing, or modifying fit-forpurpose clinical outcome assessments. Guidance for industry, food and drug administration staff, and other stakeholders. <https://www.fda.gov/media/159500/download>. Accessed Jul 18, 2022.

14. Birring SS, Prudon B, Carr AJ, Singh SJ, Morgan MD, Pavord ID (2003) Development of a symptom specific health status measure for patients with chronic cough: Leicester Cough Questionnaire (LCQ). Thorax 58(4):339–343.

15. French CT, Irwin RS, Fletcher KE, Adams TM (2002) Evaluation of a cough-specific quality-of-life questionnaire. Chest 121(4):1123–1131.

16. Yousaf N, Monteiro W, Parker D, Matos S, Birring S, Pavord ID (2010) Long-term low-dose erythromycin in patients with unexplained chronic cough: a double-blind placebo controlled trial. Thorax 65(12):1107–1110.

17. Ryan NM, Birring SS, Gibson PG (2012) Gabapentin for refractory chronic cough: a randomised, double-blind, placebo-controlled trial. Lancet 380(9853):1583–1589.

18. Vertigan AE, Kapela SL, Ryan NM, Birring SS, McElduff P, Gibson PG (2016) Pregabalin and speech pathology combination therapy for refractory chronic cough: a randomized controlled trial. Chest 149(3):639–648.

19. Vernon M, Kline Leidy N, Nacson A, Nelsen L (2010) Measuring cough severity: development and pilot testing of a new seven-item cough severity patient-reported outcome measure. Ther Adv Respir Dis 4(4):199–208.

20. Morice A, Smith JA, McGarvey L, Birring SS, Parker SM, Turner A et al (2021) Eliapixant (BAY 1817080), a P2X3 receptor antagonist, in refractory chronic cough: a randomised, placebo-controlled, crossover phase 2a study. Eur Respir J 58(5)2004240.

21. Patrick DL, Burke LB, Gwaltney CJ, Leidy NK, Martin ML, Molsen E et al (2011) Content validity--establishing and reporting the evidence in newly developed patient-reported outcomes (PRO) instruments for medical product evaluation: ISPOR PRO good research practices task force report: part 1--eliciting concepts for a new PRO instrument. Value Health 14(8):967–977.

22. Patrick DL, Burke LB, Gwaltney CJ, Leidy NK, Martin ML, Molsen E et al (2011) Content validity--establishing and reporting the evidence in newly developed patient-reported outcomes (PRO) instruments for medical product evaluation: ISPOR PRO Good Research Practices Task Force report: part 2--assessing respondent understanding. Value Health 14(8):978–988.

23. Turner-Bowker DM, Lamoureux RE, Stokes J, Litcher-Kelly L, Galipeau N, Yaworsky A et al (2018) Informing a priori sample size estimation in qualitative concept elicitation interview studies for clinical outcome assessment instrument development. Value Health 21(7):839–842.

24. US Food Drug Administration (FDA) (2022) FDA patient-focused drug development guidance series for enhancing the incorporation of the patient’s voice in medical product development and regulatory decision making. <https://www.fda.gov/drugs/development-approval-process-drugs/fda-patient-focused-drug-development-guidance-series-enhancing-incorporation-patients-voice-medical>. Accessed Mar 30, 2022.

**Abbreviations**

BA: Bachelor of Arts; BSc: Bachelor of Science; CE: concept elicitation; CI: cognitive interview; COI: concepts of interest; CSD: Cough Severity Diary; HRQoL: health-related quality of life; GCSE: General Certificate of Secondary Education; MA: Master of Arts; NHS: National Health Service; NRS: numeric rating scale; O level: General Certificate of Education Ordinary Level; PGCE: Postgraduate Certificate in Education; PhD: Doctor of Philosophy; PRO: patient-reported outcome; RCC: refractory chronic cough; SCCD: Severity of Chronic Cough Diary; SD: standard deviation; VRS: verbal rating scale.

#### Table 1 Self-reported demographics of participants

| **Characteristic** | **USA (n = 19)** | **UK (n = 10)** | **Total (*N* = 29)** |
| --- | --- | --- | --- |
| **Gender, n (%)** | | | |
| Male | 5 (26) | 1 (10) | 6 (21) |
| Female | 14 (74) | 9 (90) | 23 (79) |
| **Age (years)** | | | |
| Mean (SD) | 48 (10) | 51 (15) | 49 (12) |
| Median [range] | 49 [27–62] | 52 [23–68] | 50 [20–68] |
| **Ethnic background (USA only), n (%)** | | | |
| Hispanic or Latino | 1 (5) | — | 1 (3) |
| Not Hispanic or Latino | 18 (95) | — | 18 (62) |
| **Racial background (USA only)^a^, n (%)** | | | |
| White | 13 (68) | — | 13 (45) |
| Black or African American | 6 (32) | — | 6 (21) |
| **Racial background (UK)^a^, n (%)** | | | |
| White | — | 8 (80) | 8 (28) |
| Mixed/multiple ethnic groups | — | 2 (20) | 2 (7) |
| Black/African/Caribbean/Black British | — | 1 (10) | 1 (3) |
| **Education level (USA)^a^, n (%)** | | | |
| High school | 2 (11) | — | 2 (7) |
| Associate degree, technical, or trade school | 3 (16) | — | 3 (10) |
| Some college | 2 (11) | — | 2 (7) |
| College | 9 (47) | — | 9 (31) |
| Graduate school | 3 (16) | — | 3 (10) |
| **Education level (UK)^a^, n (%)** | | | |
| GCSE/O levels or equivalent | — | 1 (10) | 1 (3) |
| Vocational/work-based qualifications | — | 2 (20) | 2 (7) |
| University degree (BA, BSc) | — | 6 (60) | 6 (21) |
| Postgraduate degree (MA, PhD, PGCE) | — | 1 (10) | 1 (3) |
| **Residential status, n (%)** | | | |
| Living alone | 3 (16) | 4 (40) | 7 (24) |
| Living as a couple, with children | 10 (53) | 3 (30) | 13 (45) |
| Living as a couple, with no children | 4 (21) | 2 (20) | 6 (21) |
| Other^b^ | 2 (11) | 1 (10) | 3 (10) |
| **Employment status^a^, n (%)** | | | |
| Full-time employed | 12 (63) | 4 (40) | 16 (55) |
| Part-time employed | 4 (21) | 1 (10) | 5 (17) |
| Homemaker | 2 (11) | 0 (0) | 2 (7) |
| Retired | 1 (5) | 3 (30) | 4 (14) |
| Sick leave/long-term disability | 2 (11) | 1 (10) | 3 (10) |
| Other^c^ | 1 (5) | 1 (10) | 2 (7) |

^a^Not mutually exclusive.

^b^Living as a single mother with children; living with family and daughter; living with parents.

^c^Disability; freelance.

*BA* Bachelor of Arts, *BSc* Bachelor of Science, *GCSE* General Certificate of Secondary Education, *MA* Master of Arts, *O level* General Certificate of Education Ordinary Level, *PGCE* Postgraduate Certificate in Education, *PhD* Doctor of Philosophy, *SD* standard deviation

#### Table 2 Concepts spontaneously reported by participants in the CE interviews and participant example quotes

| **Concept** | **Participants reporting concept, n (%)** | | | | **Participants’ example quotes** |
| --- | --- | --- | --- | --- | --- |
|  | **Overall**  *N* = 29 | **Round 1**  n = 9 | **Round 2**  n = 10 | **Round 3**  n = 10 |  |
| **Experiences of cough with RCC** | | | | | |
| Cough characteristics | 29 (100) | ***9 (100)*** | 10 (100) | 10 (100) | *“It first starts like a tickle, like you have something in your throat, and then you just start choking.”* |
| Duration of cough | 19 (66) | ***9 (100)*** | 10 (100) | 0 (0) | *“[The cough] started, well, it would be over a year ago now, went on for weeks…It went on and on…I was just coughing the whole time.”* |
| Variability of cough frequency | 19 (66) | ***9 (100)*** | 10 (100) | 0 (0) | *“Sometimes it’s frequent and sometimes it’s not. Sometimes you may have a couple of bouts in a day and then nothing.”* |
| **Cough symptoms and symptoms related to cough** | | | | | |
| Ability to control cough | 28 (97) | ***9 (100)*** | 9 (90) | 10 (100) | *“If I start to cough, then it doesn’t stop…when it’s in full mode I have no control over that at all.”* |
| Frequency of cough | 27 (93) | **9 (100)** | 10 (100) | 8 (80) | “As the day goes on it gets more frequent, I cough more often.” |
| Coughing fits | 26 (90) | ***9 (100)*** | 10 (100) | 7 (70) | *“Once I have a coughing spell, they get closer together…but I feel like it gets out of control in a way where I feel like it’s getting worse by the second.”* |
| Severity of cough | 26 (90) | ***9 (100)*** | 10 (100) | 7 (70) | *“Well, when it’s been very bad, it’s been bad to the point of choking or vomiting.”* |
| Urge to cough | 23 (79) | ***8 (89)*** | 6 (60) | 9 (90) | *“It’s almost like a dryness in my throat…And then that’s when I cough and then it’s a constant.”* |
| Breathlessness due to cough | 16 (55) | ***3 (33)*** | 5 (50) | 8 (80) | *“Not being able to breathe…I feel like my chest is tightening or itching on the inside.”* |
| Pain due to cough | 8 (28) | ***6 (67)*** | 2 (20) | 0 (0) | *“Can be quite severe if I’ve had a proper coughing fit…My throat can be really, really sore.”* |
| Leaking urine due to cough | 5 (17) | 0 (0) | ***5 (50)*** | 0 (0) | *“Because it’s [the coughing has] been going on for so long…my bladder muscles have been affected, resulting in incontinence.”* |
| Physical discomfort due to cough | 4 (14) | ***3 (33)*** | 1 (10) | 0 (0) | *“It’s pretty uncomfortable, it’s very draining.”* |
| **Impacts and disruptions due to cough** | | | | | |
| Disruptions to social interaction | 18 (62) | ***9 (100)*** | 9 (90) | 0 (0) | *“I really don’t want to be around people because…I guess you couldn’t really socialize or anything like that because I didn’t want to be coughing non-stop in front of people.”* |
| Disruptions to daily activity | 17 (59) | ***2 (22)*** | 6 (60) | 9 (90) | *“It’s scary and it also ruins my days. If I’m planning on going someplace like going to the supermarket and if I know that that’s happening, that’s cancelled, so I’ll have to wait until another day and maybe my husband will take me.”* |
| Emotional impacts^a^ | 13 (45) | ***8 (89)*** | 5 (50) | 0 (0) | *“[I] feel really depressed and sad over it because I want to be able to do everything that other people are doing.”* |
| Disruptions to strenuous physical activities | 12 (41) | ***6 (67)*** | 6 (60) | 0 (0) | *“I actually limit my physical activity…because the more active I am, it’ll just aggravate things.”* |
| Disturbance of sleep | 25 (86) | ***9 (100)*** | 9 (90) | 7 (70) | *“I’ll have to change my sleeping position, instead of me laying down, I’ll have to prop up to try to sit up.”* |
| Waking up from sleep | 22 (76) | ***6 (67)*** | 9 (90) | 7 (70) | *“Sometimes I’ll only get 2 hours’ sleep; I’m up all night or I fall asleep and then I wake up in the middle of the night coughing.”* |
| Difficulty falling asleep | 13 (45) | ***7 (78)*** | 6 (60) | 0 (0) | *“You might just be kind of like relaxing and drifting off, and then suddenly you end up having a coughing fit.* *And it can kind of startle you, wake you up again, as well as the person lying next to you.”* |

Text in bold and italic indicates the first mention of the concept.

^a^Saturation was not reached at the symptom impact level, only at the domain level.

*CE* concept elicitation, *RCC* refractory chronic cough

#### Table 3 Participants’ understanding of individual items and revisions to the SCCD based on CI feedback

| **Item** | **Number of participants with a clear understanding of the item** | **Participants’ description of the item** | **Changes to the item stem based on CI feedback** | **Additional notes** |
| --- | --- | --- | --- | --- |
| **Cough symptoms** | | | | |
| Frequency of cough | Round 1: n = 9 (100%)  Round 2: n = 10 (100%)  Round 3: n = 10 (100%) | Described in terms of day-to-day variability and variation during the daytime versus the nighttime | None | Several participants found it difficult to remember the number of times they coughed in the past 24 hours |
| Severity of cough | Round 1: n = 9 (100%)  Round 2: n = 10 (100%)  Round 3: n = 10 (100%) | Described in terms of how bad a participant’s cough experience was, noting that severe coughing can be “debilitating” | Based on participant feedback from Round 1, the item stem wording was changed from “bad” to “severe” | — |
| Coughing fits | Round 1: n = 9 (100%)  Round 2: n = 10 (100%)  Round 3: n = 10 (100%) | Described as “episodes,” “fits,” “spells,” or “uncontrollable” or “constant” cough | Based on participant feedback from Round 1, the item stem was modified from frequency of “a coughing fit” to “coughing fits” | — |
| Urge to cough | Round 1: n = 9 (100%)  Round 2: n = 10 (100%)  Round 3: n = 10 (100%) | Described as the need or want to cough and a “tickle” in the throat | None | — |
| Ability to control cough | Round 1: n = 9 (100%)  Round 2: n = 9 (90%)  Round 3: n = 9 (90%) | Described as trying to suppress cough or attempts to lessen the frequency of cough and “calming it down” | Based on participant feedback from Round 1, the item stem was revised from the ability to “suppress cough” to “control cough” to reflect participant language | Participants indicated that their ability to control their cough was dependent on the severity and frequency of the cough |
| **Symptoms related to cough** | | | | |
| Pain due to cough | Round 1: n = 9 (100%)  Round 2: n = 10 (100%)  Round 3: n = 10 (100%) | Described in terms of frequency and severity | Based on participant feedback from Round 2, the wording of this item stem and responses for pain were revised to better indicate severity | Some participants regarded the pain and discomfort items as repetitive and felt that they were similar concepts, while others felt it was important to ask about both concepts, noting that it is possible to experience discomfort without pain |
| Physical discomfort due to cough | Round 1: n = 9 (100%)  Round 2: n = 10 (100%)  Round 3: n = 10 (100%) | Described in terms of frequency and severity | Based on participant feedback from Round 2, the wording of this item stem and responses for discomfort were revised to better indicate severity |  |
| Breathlessness due to cough | Round 1: N/A  Round 2: N/A  Round 3: n = 10 (100%) | Described in terms of an inability to complete daily tasks due to difficulty catching breath | None | — |
| Leaking urine due to cough | Round 1: N/A  Round 2: n = 10 (100%)  Round 3: n = 10 (100%) | Described as involuntarily “wetting yourself” or “passing urine” when coughing | None | A male participant mentioned that this item was less relevant to men and probably more applicable to women |
| **Disruption to activities due to cough** | | | | |
| Disruptions to usual household activities | Round 1: n = 9 (100%)  Round 2: n = 10 (100%)  Round 3: n = 10 (100%) | Described as doing laundry, cleaning the house, or gardening | After Round 1, this item stem wording was changed from “usual activities” to “usual household activities” for clarity and differentiation from the new separate item about disruptions to strenuous activities | — |
| Disruptions to strenuous physical activities | Round 1: N/A  Round 2: n = 10 (100%)  Round 3: n = 10 (100%) | Described as exercise and housework requiring more physical effort | None | — |
| Disruptions to social interaction | Round 1: N/A  Round 2: n = 10 (100%)  Round 3: n = 10 (100%) | Described as meeting and speaking to other people | None | — |
| **Disruption to sleep due to cough** | | | | |
| Difficulty staying awake during the day due to coughing the night before | Round 1: N/A  Round 2: n = 8 (80%)  Round 3: n = 10 (100%) | Described as “drifting off,” “feeling drowsy,” and “not feeling refreshed” | Given the difficulty experienced by some participants in attributing tiredness during the day to coughing at night, the item stem was reworded for Round 3 to ensure attribution was made to cough. The item was deleted from Version 1.0 of the SCCD as the instrument development team found the concept could be influenced by factors such as comorbid conditions (e.g., obstructive sleep apnea) and would also be inappropriate for night workers or people who sleep during the day | Participants mentioned that it was difficult to know the reason they had had disturbed sleep. One participant responded to the item based on their difficulty sleeping at night, rather than their difficulty staying awake during the day, and another participant suggested deleting the item as it was a bit lengthy and complicated |
| Difficulty falling asleep | Round 1: n = 9 (100%)  Round 2: n = 10 (100%)  Round 3: n = 10 (100%) | Described as not being able to go to sleep due to coughing and taking longer to fall asleep | After Round 1 this item stem wording was modified from “trouble” to “difficulty” in order to emphasize how participants viewed the concept | — |
| Disturbance of sleep | Round 1: n = 9 (100%)  Round 2: n = 10 (100%)  Round 3: n = 10 (100%) | Described as interrupted sleep, poor sleep quality, or being woken up due to coughing | None | Wording revisions were made by the translatability team |
| Waking up from sleep | Round 1: n = 7 (78%)  Round 2: n = 10 (100%)  Round 3: n = 10 (100%) | Participants described times when they woke up at night coughing | Participants’ feedback indicated that this concept was captured by “sleep disturbed by cough” and a decision was made by the instrument development team to delete the item from Version 1.0 of the SCCD due to redundancy | Participants preferred the item on sleep disturbance as it was more descriptive of their experience |

*CI* cognitive interview, *N/A* not applicable, *SCCD* Severity of Chronic Cough Diary

#### Figure legends

#### Fig. 1 Overview of the steps involved in the development of the SCCD.

^a^This article focuses on Stage 4: assessing the content validity of the SCCD. The preceding Stages 1–3 of SCCD development are briefly described in the Supplementary Information.

^b^Stage 2 clinical expert interviews were conducted with three clinicians who treat patients with RCC in order to understand the clinical practice experience of symptoms of RCC and to assess the concepts of interest identified in the literature review concepts. Relevant cough severity concepts were obtained and informed the development of the patient interview guide.

^c^Clinical expert feedback from one of the three clinicians in Stage 2 was also obtained on the SCCD Version 0.1 and Version 0.3 to assess the clinical relevance of the concepts included in the SCCD before the patient interviews.

*CE* concept elicitation, *CI* cognitive interview, *RCC* refractory chronic cough, *SCCD* Severity of Chronic Cough Diary

#### Fig. 2 Revised conceptual framework of the SCCD Version 1.0 following qualitative research

^a^Important for women only.

*RCC* refractory chronic cough, *SCCD* Severity of Chronic Cough Diary
